# Inactivated Viral Vaccine BBV87 Protects Against Chikungunya Virus Challenge in a Non-Human Primate Model

**DOI:** 10.3390/v17040550

**Published:** 2025-04-10

**Authors:** Sarah L. Kempster, Deborah Ferguson, Claire Ham, Joanna Hall, Adrian Jenkins, Elaine Giles, Simon L. Priestnall, Alejandro Suarez-Bonnet, Pierre Roques, Roger Le Grand, Sumathy Kandaswamy, Sushant Sahastrabuddhe, Libia Milena Hernandez, Sunee Chuasuwan, Hyeon Seon Ahn, Deok Ryun Kim, Anh Wartel, Raphaël M. Zellweger, Neil Berry, Neil Almond

**Affiliations:** 1 Science and Research, Diagnostics, Medicines and Healthcare Products Regulatory Agency, Blanche Lane, South Mimms EN6 3QG, Hertfordshire, UK; sarah.kempster@mhra.gov.uk (S.L.K.);; 2 Department Pathobiology & Population Sciences, The Royal Veterinary College, Hawkshead Lane, North Mymms, Hatfield AL9 7TA, Hertfordshire, UK; spriestnall@rvc.ac.uk (S.L.P.); asuarezbonnet@rvc.ac.uk (A.S.-B.); 3 INSERM, CEA, Center for Immunology of Viral, Auto-Immune, Hematological and Bacterial Diseases (IMVA-HB/IDMIT), Université Paris-Saclay, 92260 Fontenay-aux-Roses, France; pierre.roques@cea.fr (P.R.);; 4 Bharat Biotech International Ltd., Hyderabad 500078, India; 5 International Vaccine Institute (IVI), Seoul 08800, Republic of Korea; sushants@ivi.int (S.S.); libia.hernandez@ivi.int (L.M.H.);

**Keywords:** chikungunya virus, vaccine, non-human primate, pathogenesis, immunity

## Abstract

Chikungunya virus (CHIKV) is an alphavirus transmitted by mosquitos that poses a threat to global public health and for which there is an urgent need for widespread access to globally licensed vaccines. Here, we demonstrate that an inactivated CHIKV vaccine (BBV87) protects against systemic infection with CHIKV in a non-human primate (NHP) challenge model. Groups of five cynomolgus macaques received two doses of 20 µg BBV87 vaccine or saline alone (28 days apart). Twenty-eight days after the second immunisation, all animals were challenged with CHIKV. All controls were productively infected with detectable viremia and pathological responses following challenge, including altered thermoregulation, haematological and cytokine changes. Critically, the histopathological analysis of finger joints identified areas of inflammation in the synovium. By contrast vaccinated macaques had no detectable viremia and none of the pathological changes were reported in control animals. This study demonstrates that a 20 µg dose of BBV87 vaccine confers robust protection in vivo, both on the acquisition of infection and pathology.

## 1. Introduction

Chikungunya virus (CHIKV) has a high global burden with long-term polyarthralgia sequelae being the predominant morbidity [1]. CHIKV is a positive-sense RNA virus belonging to the alphavirus genus of Togaviridae with four lineages: Asian urban (AUL), East Central and South African (ECSA), Indian Ocean (IOL) and West African (WA) [2]. Although multiple genotypes exist for CHIKV, it is generally accepted there is only one serotype [3,4]. Following the outbreak in the Indian Ocean region that began in 2005 [5], the global spread of CHIKV has increased, resulting in epidemics on most inhabited continents in tropical and sub-tropical regions [6]. CHIKV is transmitted by Aedes spp. mosquitoes that cause acute febrile illness sometimes followed by long-term severe and debilitating joint pain [7]. Although rarely fatal, the effects of virus infection impact significantly on individual health with related socio-economic implications. Relatively recent and large epidemics have characterised the rapid spread of CHIKV in India and the Indian Ocean region, and established CHIKV as a global health concern reinforced by spread of the virus into the Western hemisphere, a region with no pre-existing immunity [8].

With no therapeutics available for the specific treatment of CHIKV infection there is currently an urgent need for widespread access to globally licensed vaccines. Currently there is only one licensed vaccine: a live attenuated virus. Due to the epidemic character of CHIKV, a phase III efficacy study with vaccine candidates is considered very challenging [9]. Therefore, the first vaccine was licensed on the basis of clinical immunogenicity and animal challenge studies. As a consequence, pre-clinical immunisation and challenge experiments are important to generate pro-forma efficacy data as a crucial component of any licensure dossier. Non-human primates (NHPs), particularly cynomolgus macaques, have been shown to be a suitable infection model for assessing vaccine candidates against CHIKV as they exhibit pathology as well as virological and immunological responses following infection, similar to disease in humans [10,11,12,13]. As a preliminary study, we evaluated whether an experimental CHIKV vaccine candidate in clinical trial evaluation (BBV87 from Bharat Biotech International Limited) would protect in a NHP challenge model system, when delivered in an identical dose and timetable as in early clinical evaluation. The study schedule demonstrated that active immunisation with this vaccine confers potent protection in this model. Furthermore, it validated the use of this challenge model to evaluate antisera generated from clinical evaluation of this vaccine, as has been applied previously to the existing licensed vaccine.

## 2. Materials and Methods

### 2.1. Challenge Agent Chikungunya Virus LR2006 OPY1

The LR2006 OPY1 virus is a well-characterized strain of CHIKV that was originally isolated from the serum of a febrile French patient returning from La Réunion Island [14]. The CHIKV (LR2006 OPY1 strain, GenBank: DQ443544) challenge stock derived from passage on Vero cells was provided by Roger Le Grand’s laboratory (CEA, Paris) [15]. This strain has been previously used for NHP challenge models by others [16]. To confirm the challenge stock used in these studies did not contain any deletions or mutations relative to the published sequence, whole genome deep sequencing analysis was performed directly using four overlapping amplicons spanning the CHIKV genome and subjected to Illumina sequencing using overlapping primer sets (Supplementary Table S1). A four primer pair strategy was adapted to amplify the entire region of the chikungunya virus genome (~12 kB, GenBank: DQ443544) as described by Mohamed Ali et al. [17]. Trimmed reads were analysed using the iVar bioinformatics programme.

### 2.2. Challenge Model

Purpose-bred cynomolgus macaques, from a UK-based colony, were allocated to groups (2 females and 3 males per group, age range of 3 years 9 months to 4 years 10 months) by randomisation. All procedures were carried out in a UK Designated Establishment under Home Office License in accordance with the Animal Scientific Procedures Act 1986. Group sizes of 5 were calculated by applying Fisher’s Exact Test to ensure a statistically significant result at the 5% level and provide resilience in the experimental outcome.

### 2.3. Surgical Implants

Telemetry implants (model 201, EMMS, Hampshire, UK) were inserted surgically between the first and second immunisations. Implants were positioned subcutaneously without tethering on the back to the right (subjects left) of the midline, level with the lower end of the shoulder blade and subjects were allowed to recover for at least 7 days before further procedural work. The protocol for surgery included sedation for handling with 8 mg/kg i.m. Narketan and the maintenance of anaesthesia by gaseous Isoflurane (2%). Blood pressure and heart rate were monitored throughout surgery and post-surgery oxygen delivery. Atropine (0.05 mg/kg i.m.) was administered to reduce saliva and fluid in the respiratory tract. Metcalm (0.2 mg/kg, s.c) and Naxcel (5 mg/kg, i.m.) were administered for pain relief and wound infection control, respectively. Marcaine was administered topically (0.25%) during surgery for local anesthetic at the surgery site. No adverse health observations or issues were observed as a result of surgery or anaesthesia, and no other veterinary interventions were required throughout the study. The implanted telemetry device transmitted wirelessly to receiving antennae within the room every 30 s. From these data, the average subcutaneous temperature for each individual per hour was calculated. A random effects mixed model, in which the residual covariance structure assumes a first-order autoregressive, incorporating the diurnal rhythm of temperature, was applied to the implant data to identify significant differences between vaccinees and controls [18]. Analyses were performed using SAS Proc Mixed (v9.4, SAS, Cary, NC, USA).

### 2.4. Vaccine

BBV87 is a CHIKV vaccine prepared as inactivated whole virion preparation from the CHIK/03/06 strain derived from an Indian (2006) isolate of the East, Central and South African (ECSA) genotype, and propagated on Vero cells [19]. The resulting virus preparation was inactivated by beta propiolactone, an organic compound widely used as an inactivating agent in vaccine production. Inactivation was considered complete when no infectious particles were detected after three rounds of propagation in vitro for 7 days on permissive cells. Infectious virus particles were enumerated in a plaque assay at the end of three serial rounds of amplification. The validated plaque assay has a detection limit of 1 virus particle. The vaccine was formulated with 0.25 mg of aluminium hydroxide as adjuvant.

### 2.5. Experimental Outline (See Figure 1)

**Figure 1:**
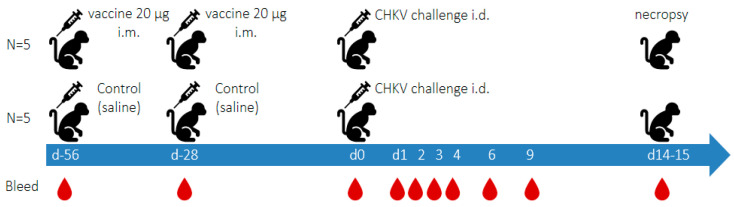
Study outline showing vaccination and bleed intervals for both vaccinated and control animals.

Two 20 µg doses of the vaccine candidate BBV87 were administered to five cynomolgus macaques 28 days apart via the intramuscular route (right then left leg, rectus femoris). This vaccination regimen has been used in clinical trials. A second group of 5 cynomolgus macaques received saline (0.9% sodium chloride) at the same time intervals to serve as controls. No adverse effects were observed after the administration of vaccine doses 1 or 2 in any animals. Twenty-eight days after the second vaccination, animals were challenged intra-dermally (right arm, deltoid) with 1 × 10^5^ PFU CHIKV (LR2006 OPY1 strain) and monitored daily before termination on days 14 or 15 post-challenge. Cynomolgus macaques were bled periodically before and after immunisation and challenge, as outlined in Figure 1. Blood was kept unprocessed (whole blood) or separated into serum or plasma. Haematological analyses were performed with EDTA-treated blood samples using a Pentra 60 C+ haematology analyser (Horiba, Northampton, UK). The isolated sera and plasma were stored frozen at 80 °C until testing by RT-qPCR or culturing for virus (only one freeze thaw was performed).

### 2.6. Histopathology

Samples at necropsy joints (knuckles) from the right hand and major organs (liver, draining lymph node, spleen, and brain) were taken for analyses. Samples were placed into 10% neutral buffered formalin for histological analyses or snap frozen for molecular analyses. One intact right hand per animal was fixed at room temperature for 2 weeks in 10% neutral buffered formalin prior to dissection into individual finger (Thumb, F1–F4) and knuckle (Thumb Kn, F1Kn–F4Kn) joints (maximum of 10 samples per animal). These joints were decalcified (minimum 1 week) using multiple changes of decalcification solution (15% EDTA/0.1M Tris-HCl, pH 7.6). Decalcification was determined to be complete following two sequential negative results for the presence of calcium in the decalcification solution. The presence of calcium was determined by combining an aliquot of decalcifying solution from each joint with 0.2 M citric acid/0.16 M dibasic potassium phosphate solution (pH 3.2–3.6) and 5% saturated ammonium oxalate solution. The lack of a visible precipitate in solution on mixing indicated sufficient decalcification. Joints were processed into paraffin blocks using standard histological protocols and 4 µm sections from each joint haematoxylin and eosin stained using standard histological protocols. Inflammatory responses in and around synovial joints and any associated joint deterioration for 10 joints per animal (or maximum available) were assessed independently by two veterinary pathologists.

### 2.7. Plaque Assay for Determination of Viral Load in Blood and Selected Tissues

Tissue samples stored at −80 °C (approximately 20 mg) were homogenised in virus transport media (VTM, Hanks balanced salt solution with 2% heat-inactivated foetal calf serum (HI-FCS), penicillin/streptomycin, 0.5 µg/mL amphotericin B) by three consecutive rounds of bead beating (TissueLyserLT, Qiagen, Manchester, UK) at 50Hz for 30 s followed by 1 min rest on ice. Vero cells maintained in DMEM with 10% HI-FCS and antibiotics (penicillin, streptomycin) were seeded in 96-well plates at 3 × 10^4^/well and infected the following day with a freshly prepared serial dilution of the test sample (tissue homogenate or serum). Each sample only had one freeze thaw before virus testing by culture. After 2 h the supernatant was removed and 200 µL of media (DMEM, 4% HI-FCS) was added to each well. Cells were incubated for 3 days before monolayers were fixed with 4% paraformaldehyde and plaques visualised by staining with 0.2% (*w*/*v*) methyl violet. Plaques and the cytopathic effect were recorded to calculate the virus titre using the Reed–Muench method [20].

### 2.8. Quantitative CHIKV RT-qPCR Determinations in Plasma and Tissues

Quantitative analyses of CHIKV-specific viral RNA were performed on plasma and selected lymphoid tissues. Tissues (20 mg of spleen, mesenteric or peripheral lymph nodes) were homogenised using a TissueLyserT (Qiagen) with 5-mm steel beads in 300 µL DMEM at 50 Hz for 2 min. This was followed by the addition of 300 µL MagnaPure 96 Extraction Lysis Buffer and a further 1 min of homogenisation. The sample was then filtered through a 50 µL mesh (Filcon, BD Biosciences, Wokingham, UK). For plasma, 200 µL was lysed with 250 µL MagnaPure 96 External Lysis buffer. Both plasma and tissue samples were subjected to extraction using the Roche, MagnaPure 24 extraction platform using Nucleic Acid extraction cartridge with an off-board lysis and plasma protocol with a final elution into 50 µL. CHIKV RT-qPCR was performed with 5 µL of nucleic acid extract with forward (5′-AAGCTYCGCGTCCTTTACCAAG-3′) and reverse (5′-CCAAATTGTCCYGGTCTTCCT-3′) primers and probe (5′-FAM-CCAATGTCYTCMGCCTGGACACCTTT-BHQ1-3′) with RT conditions of 56 °C for 30 min followed by 5 min 95 °C and 50 cycles of amplification: 95 °C 15 s, 95 °C for 1 min. Each sample was tested in triplicate and viral loads are expressed in International Units (IU), relative to the International Standard for CHIKV RNA [21]. The lower limit of detection was established to be 50 CHIKV RNA IU/mL plasma and 20 CHIKV RNA IU/100 ng total RNA for tissues.

### 2.9. Binding Antibody ELISA

Commercially available CHIKV IgG and IgM ELISAs (EI 293a-9601 G and EI 293a-9601 M, respectively, EuroImmun, Lübeck, Germany) cross-react with cynomolgus macaque antibodies and were used to quantify CHIKV antibodies after each vaccination (d-56 and d-28) pre-challenge (d0) and at 9 and 14 days post-infection. The assay was performed in accordance with the manufacturer’s instructions.

### 2.10. CHIKV Plaque Reduction Neutralisation Test

Neutralising antibodies were assessed using a plaque reduction neutralisation test (PRNT). Cynomolgus macaque sera were heat-treated at 56 °C for 30 min and diluted in virus diluent (DMEM). A total of 50 µL of a 2-fold serial dilution of serum samples was mixed with an equal volume of CHIKV OPY1 (target of 100 i.u./well) and incubated for 1 h at 37 °C prior to the addition to Vero cells that had been seeded at 3 × 10^4^/well in 96-well plates one day prior. After 2 h, well contents were removed and 200 µL of media (DMEM, 4% HI-FCS) was added to each well and cells were incubated for 3 days before fixation with 4% paraformaldehyde and subsequent visualisation of plaques by staining cells with 0.2% (*w*/*v*) methyl violet. Wells were either scored as positive or negative based on whether plaques were observed. The neutralising antibody titre was calculated as the reciprocal of the highest serum dilution that reduced the number of virus plaques in the test by 50% [20].

### 2.11. Multiplex Cytokine Assay

Serum was tested in the cytokine multiplex assay based on the Luminex technology (Milliplex Non-Human Primate Cytokine/Chemokine MAGNETIC Bead Premixed 23 Plex Panel, cat no: PCYTMG-40K-PX23, Merck, Gillingham, UK) for levels of selected cytokines G-CSF, GM-CSF, IFN-g, IL-1ra, IL-1b, IL-2, IL-4, IL-5, IL-6, IL-8, IL-10, IL-12/23 (p40), IL-13, IL-15, IL-17, IL-18, MCP-1, MIP-1a, MIP-1b, sCD40L, TGF-a, TNF-a and VEGF, at days 0, 2, 4, 6 post-challenge. The assays were performed according to the manufacturer’s instructions. Sample cytokine concentrations were calculated from the reference standards included in each assay. Statistical significance was determined by ANOVA with post-hoc Bonferroni. C-reactive protein (CRP) serum levels were measured using the Monkey CRP ELISA Kit (ab260062, Abcam, Cambridge, UK) according to the manufacturer’s instructions.

## 3. Results

### 3.1. Virus Sequence of the Challenge Stock

Sequencing of the original stock used for challenge studies was undertaken to assure the fidelity of challenge virus material. Whole genome sequencing generating >20-fold coverage by Illumina technology identified only minor sequence changes mapped to the chikungunya virus reference strain (LR2006 OPY1) (Table 1). Of note is a 6% occurrence of the Opal stop mutation to arginine that is recognised as a mutation in alphaviruses. Secondly, a 17% occurrence of G82R, that is recognised as an attenuating mutation, is also noted.

### 3.2. Serological Responses to BBV87 Vaccination and CHIKV Infection

**Figure 2:**
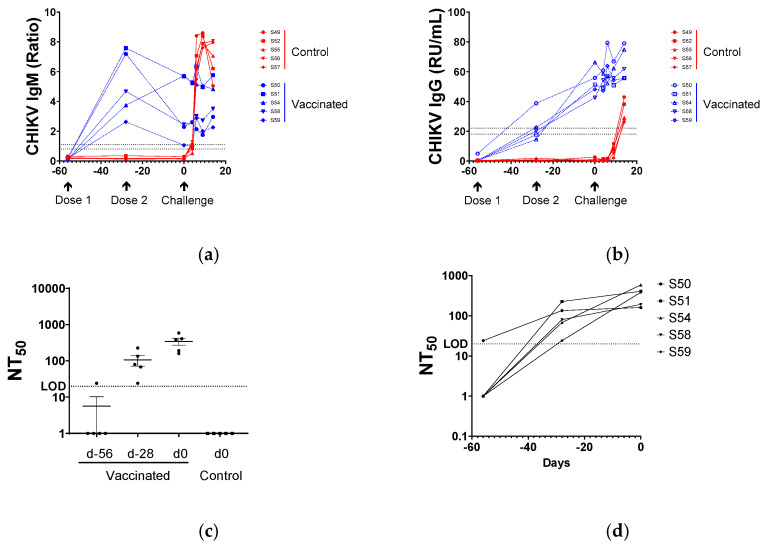
Anti-CHIKV antibody responses: (**a**) IgM antibody responses detected by ELISA following vaccination with BBV87 (blue) or control (red), and subsequent challenge with virus. The horizontal dotted lines denote the assay manufacturer’s interpretation of results criteria. Below the lower dotted line at Ratio 0.8 is considered negative, between the dotted lines (0.8–1.1 Ratio) is interpreted as equivocal, and >1.1 Ratio as positive. (**b**) IgG detected by ELISA following vaccination with BBV87 (blue) or control (red), and subsequent challenge with virus. The horizontal dotted lines denote the assay manufacturer’s interpretation of results criteria. Below the lower dotted line at 16 RU/mL is considered negative, between the dotted lines (16–22 RU/mL) is interpreted as equivocal, and >22 RU/mL positive. (**c**) In vitro neutralisation titres of vaccinated and control animals post-first and -second vaccination, and at time of challenge. (**d**) In vitro neutralisation of each vaccinated individual over time. LOD denotes limit of detection of neutralisation assay.

Anti-CHIKV IgM was detected in sera by ELISA 28 days after a single BBV87 immunisation. Levels generally declined following the second immunisation and remained at steady levels following the virus challenge. Controls had no detectable CHIKV IgM antibodies on the day of the virus challenge. All control macaques seroconverted with IgM antibodies detectable by ELISA by day 6 post-virus challenge, and levels remained high through to the end of the study at day 14 (Figure 2a). Following immunisation with BBV87, anti-CHIKV IgG responses were detected in the serum of all vaccinated animals by ELISA, at 28 days post-immunisation. IgG levels increased following the second immunisation in serum taken at the time of the challenge (Day 0). At the time of the challenge, all BBV87-immunised macaques were anti-CHIKV sero-positive. No specific serological responses were detected in control macaques at the time of the challenge. Following the virus challenge, a small and increasing level of anti-CHIKV IgG was detected in the serum of BBV87-immunised individuals. By contrast, control macaques seroconverted showed a rapid increase in anti-CHIKV IgG responses by day 14 post-challenge (Figure 2b).

### 3.3. Virus Neutralisation

Prior to the first immunisation (day-56), four of five macaques had no detectable CHIKV neutralising activity in the serum, whereas the serum sample of one macaque (S50) had sero-reactivity at the limit of detection (LOD 1:20 dilution). Twenty-eight days after the first immunisation (day-28), serum from all vaccinated animals was able to neutralise CHIKV, and neutralising activity was boosted with the second immunisation. On the day of the virus challenge, the range of the neutralisation titre was 160–586 NT_50_. For animal S50, which had marginal neutralising activity prior to immunisation, serum neutralising activity rose more than five-fold by day 28, and by a further 0.2-fold by the day of challenge (day 0), which is a similar level to that seen in other vaccinees. No serum-neutralising activity was detectable in the serum of controls on the day of the virus challenge (Figure 2c,d).

### 3.4. Virus Detection in Blood Post CHIKV Challenge

**Figure 3:**
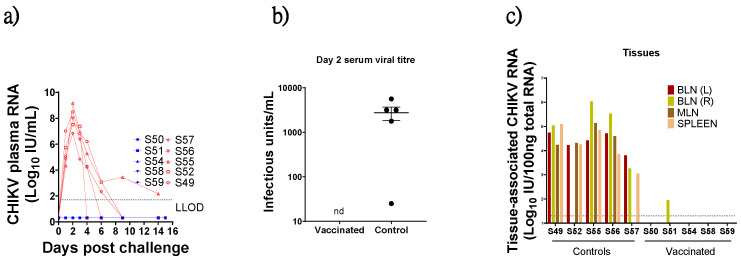
(**a**) CHIKV RNA as measured by RT-qPCR in plasma of macaques that had been immunised with BBV87 (blue) or saline prior to virus challenge (red). Lower limit of detection (LLOD) denoted by dotted line. (**b**) CHIKV infectious units/mL in serum of vaccinated and control animals two days post-challenge (mean ± SEM). Negative samples were assigned an arbitrary value of 2, nd = not detected. (**c**) CHIKV RNA in each individual animal in (left to right): left brachial lymph node (BLN(L)), right brachial lymph node (BLN(R)), mesenteric lymph node (MLN) and spleen expressed as CHIKV RNA International Units (IU)/100 ng total RNA.

Viral RNA was detectable by RT-qPCR in the plasma of all control macaques on day 1 following challenge, peaking on day two post-challenge (range 7–9 log10 CHIKV RNA IU/mL) before falling markedly to below detectable levels (<50 CHIKV RNA IU/mL) in four of five macaques by day nine post-challenge. One macaque (S55) exhibited persisting viral RNA levels up to the end of the study on 14 days post-challenge. By contrast, viral RNA was not detectable in plasma from BBV87-immunised macaques at any time following virus challenge (Figure 3a). At day two this difference was highly significant (Mann–Whitney test, *p* = 0.008). Live virus was recovered from serum of all control animals two days post-challenge but not from immunised animals (Figure 3b). Notably, S57 had the lowest viral titre by RT-qPCR and the lowest level of recoverable virus in serum.

### 3.5. Virus Detection in Tissues

At termination (14/15 days post-challenge), tissue-associated viral RNA was detected in 18 of 20 lymphoid tissues sampled (spleen, mesenteric or either left or right brachial lymph node (LN) from control animals and at relatively high (>4 log10 IU CHIKV RNA/100 ng total RNA) levels, except S57 which had lower tissue RNA levels coincident with the lower plasma RNA and infectious virus recovered in this macaque (Figure 3c). By contrast, for all immunised individuals, CHIKV-specific signal was detected in only one tissue from one animal (Right brachial LN of vaccinated S51). To investigate whether the viral nucleic acid detected by RT-qPCR methods was infectious, frozen and homogenised tissue samples were defrosted and co-cultured with permissive cells. No infectious virus was recoverable from homogenised spleen, brachial lymph node (left or right) or ILN-PLN from any animals irrespective of immunisation protocol.

### 3.6. Temperature Monitoring

**Figure 4:**
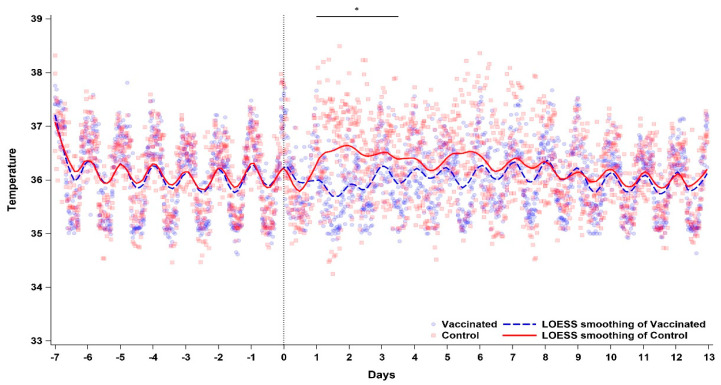
Temperature profiles of BBV87-immunised (blue) and control (red) macaques before and after CHIKV challenge at time 0 (represented by dotted line). Lines represent LOWESS (Locally Weighted Scatterplot Smoothing) smoothed means of the groups, and points represent individual readings. Asterisk denotes time of significant increase in temperature in control group compared to vaccinated group from 24 to 84 h post-challenge (*p* < 0.05).

Prior to virus challenge, a robust and regular diurnal pattern of temperature change was observed in all macaques. Following virus challenge, this pattern was disrupted in all individuals that received the placebo, notably between 24 and 84 h (1–3.5 days) after challenge, and then 120–144 h (5–6 days) post-challenge. No observable interruption of diurnal pattern was observed in individuals vaccinated with BBV87. Statistical analyses showed a significant difference between the vaccinated and control groups from 1 to 3.5 days post-challenge (*p* = 0.0425) (Figure 4).

### 3.7. Haematology

**Figure 5:**
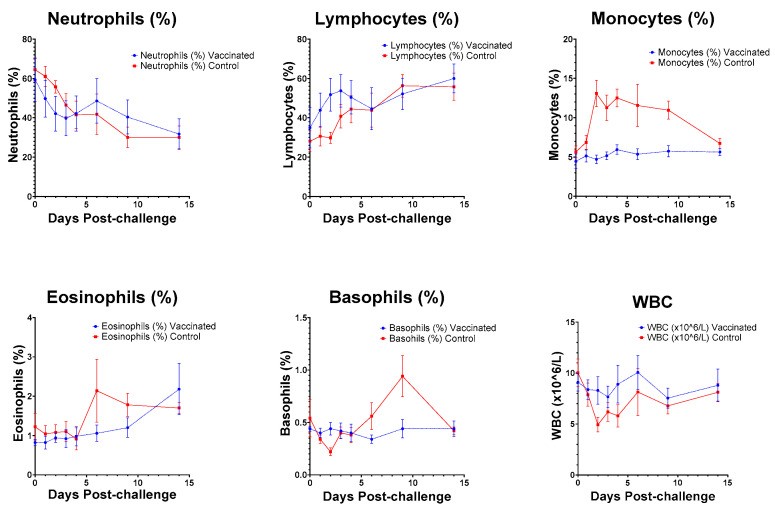
Haematological changes observed in BBV87-immunised (blue circles with dashed line) and controls (red squares with solid line) following challenge with CHIKV. Means ± SEM.

Five-part haematology analyses were performed on blood samples. Between days two and six, post-virus challenge total WBC counts were lower in controls compared to in vaccinated macaques. This trend belied an increase in lymphocytes in BBV87-vaccinated macaques between days 1 and 6 and a marked increase in monocytes in placebo-treated macaques on days 2–14 (Figure 5). No perturbation of clinical chemistry markers was observed in any individual (Supplementary Table S2).

### 3.8. Joint Pathology

**Figure 6:**
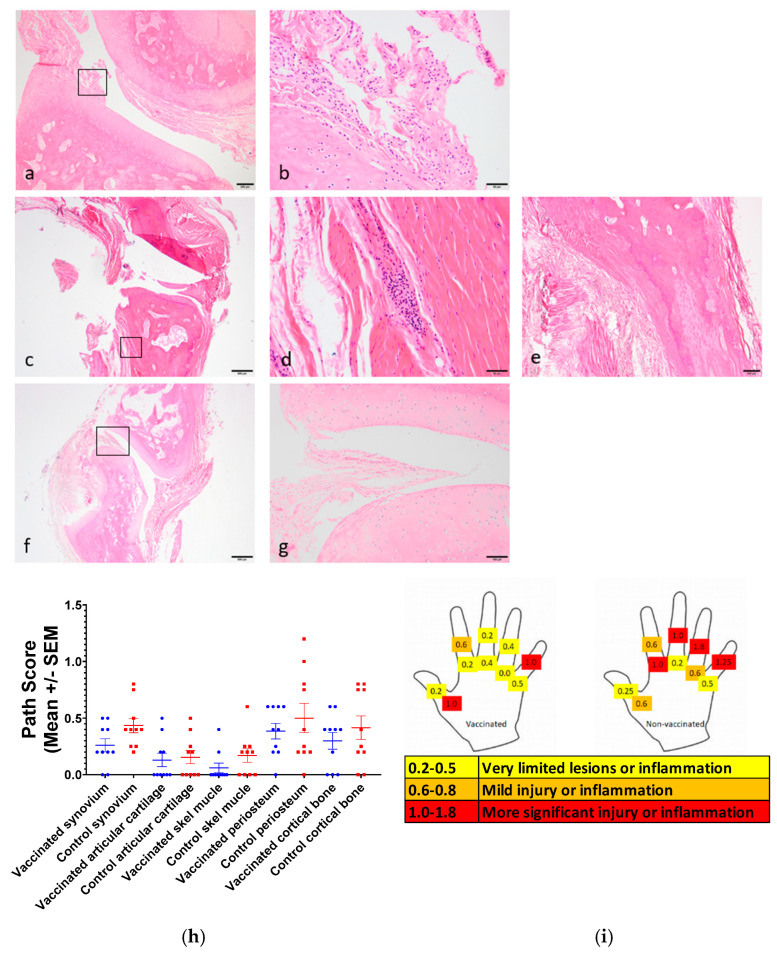
Representative images of pathology present within the finger joints of non-vaccinated (**a**–**e**) and vaccinated (**f**,**g**) cynomolgus macaques at termination (14–15 days post-challenge). (**a**) Lymphocytic and plasma cell infiltrate within synovium (scale bar = 200 µm), (**b**) higher magnification of boxed area of joint articulating surfaces with lymphocytes and plasma cells widely distributed within the synovium indicating early events in the development of an arthritic response (scale bar = 50 µm). (**c**–**e**) Lymphocytic and plasma cell inflammatory infiltrates present within skeletal muscle indicative of viral aetiology ((**c**): scale bar = 500 µm and boxed area shown in (**d**), scale bar = 50 µm, (**e**) scale bar = 500 µm). (**f**) Joint from vaccinated animal with no inflammatory pathology present (scale bar = 500 µm). (**g**) higher magnification of boxed areas of joint articulating surfaces showing a normal synovium (scale bar = 100 µm. (**h**) Points represent the scores for each individual animal; for each pathology parameter, lines represent mean ± SEM, (**i**) schematic representation (finger and location) of inflammatory changes in the right-hand knuckle or finger joints from vaccinated (s50, left hand image) and control animals (right hand image).

No animals showed any observable clinical signs associated with CHIKV challenge. Histologically, control animals exhibited subtle but distinct inflammatory changes within the joint synovium, articular cartilage and skeletal muscle surrounding joints two weeks after challenge with CHIKV. Representative images are shown in Figure 6. Inflammatory infiltrates (Figure 6a–e) comprised lymphocytes and plasma cells consistent with the tissue disease of viral aetiology. Irregularities developing on the surface of some articulating joints (Figure 6a,b) and the presence of inflammation and thickened synovium suggested early stages of arthritis. Full pathology results are presented in Supplementary Table S3. In immunised, virus-challenged animals, baseline levels of pathology were present in some joints, but these levels were lower than those observed in joints from controls. Furthermore, a greater number of joints exhibited no evident histopathology (Figure 6f,g). The assessment of pathology described above is represented for each joint and location (Figure 6h), and as a mean score for each parameter (Figure 6i).

### 3.9. Cytokine Analyses

**Figure 7:**
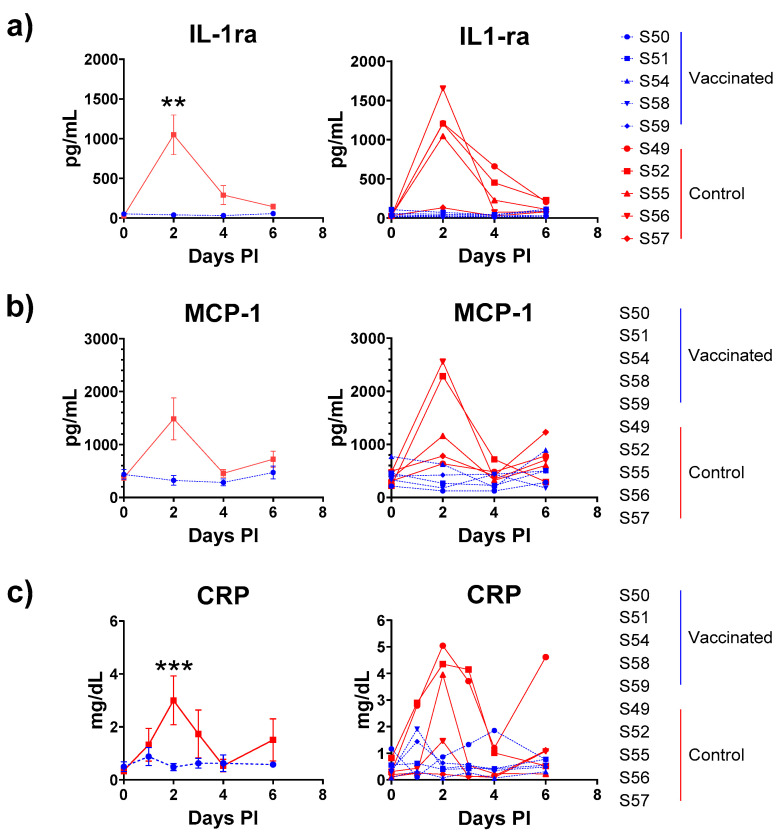
(**a**) IL-1ra, (**b**) MCP-1 and (**c**) CRP levels post-challenge in vaccinated (blue with dashed lines) and control (red with solid lines) animals plotted as mean ± SEM (left panels) and as individual plots (right panels). Statistical significance determined by Anova test with Post-hoc Bonferroni (*** *p* < 0.01, ** *p* < 0.05).

Cytokines were determined at the time of challenge, and two, four and six days post-challenge using a multiplex assay. Of the 23 cytokines measured, only IL-1ra was significantly elevated at two days post-challenge in controls compared with immunised animals (Figure 7a). An increase in MCP-1 was observed in control challenged macaques, but the difference did not achieve significance at the 5% level (Figure 7b). For the single-plex CRP ELISA, control animals had a significantly elevated level of CRP in serum two days post-challenge compared with vaccinated animals (Anova *p* = 0.0099, post-hoc Bonferroni *p* < 0.01) (Figure 7c). Of note was the observation that the lowest CRP levels in the serum were observed in control S57 that exhibited the lowest viraemia.

## 4. Discussion

The widespread availability of licensed vaccines, effective against CHIKV infection and associated disease, would significantly impact populations where outbreaks are likely to occur. In addition, it would reduce global distribution and frequency of significant outbreaks by breaking the transmission cycle, especially in urban settings. However, the episodic and unpredictability of CHIKV outbreaks means that designing and conducting conventional phase III trials to assess candidate vaccine efficacy remains challenging [26]. In the absence of clinical trial efficacy data, studies in pre-clinical models are crucial to complement immunogenicity data from clinical trials and support regulatory submissions. Both the immunogenicity and efficacy of candidate vaccines may be evaluated in NHP challenge models for assessing both protection against infection and pathogenesis associated with primary CHIKV exposure in naïve hosts.

In this study, the potential of an inactivated candidate prophylactic vaccine for CHIKV that has completed two Phase I clinical trials (Clinical Trials Registry of India ref: CTRI/2017/02/007755 and CTRI/2020/04/024533) was evaluated in Indonesian cynomolgus macaques. The animals were immunised twice with 20 µg of the candidate vaccine (BBV87) or a placebo (0.9% sodium chloride) 28 days apart to mirror the vaccination protocol in clinical trial volunteers. Twenty-eight days after the second immunisation, all the macaques received intradermal challenge with a high dose of CHIKV. Post-challenge virology, immunology and pathology data were collected to allow the robust interpretation of vaccine efficacy. The data demonstrated that this vaccine regimen was able to confer potent protection against this robust virus challenge both in terms of its impact on virus replication and host pathology.

Immunisation with BBV87 vaccine elicited marked CHIKV-specific binding and neutralising antibody responses, which were boosted by the second immunisation. CHIKV-specific IgM responses were observed in all vaccinated animals after both vaccinations. This sustained level of IgM in vaccinees may have been due to the cross-reactivity of IgG in the IgM ELISA as the initial step involves an IgG neutralisation procedure that can be saturated in the presence of high levels of IgG. It was surprising to detect evidence of very low levels of neutralizing seroactivity in one of the vaccinates prior to immunization. This is highly unlikely to be specific anti-chikungunya virus seroreactivity but a non-specific cross-reactivity, since the macaques used in this study were from a closed breeding colony based in the UK where the chikungunya virus is not found. Furthermore, following immunization the subsequent kinetics of specific neutralizing seroreactivity were identical with the other members of this treatment group, which would not have been the case if this individual had been specifically primed. LR2006 OPY1 is a relevant virus isolate to evaluate vaccine efficacy, as it was reisolated from travellers returning from Indian Ocean Islands of La Reunion and Mayotte (French territories) in 2005 as described by Parola et al. [15]. The sequencing of the stock used for challenge in this study confirmed that it had not undergone significant changes in sequence integrity since the first description of this isolate. Two sequence changes in the viral stock compared with the reference strain merited further interest, firstly a 6% minority population of the opal stop mutation in nsp3 to an arginine (R) residue (aa:5645) was identified—this mutation occurs in many alphaviruses and CHIKV isolates naturally, such as in SGP007, a CHIKV isolate from a patient in Singapore [27]. Others have shown that the absence of the opal stop reduces infectivity in vitro and in the mouse model, and has been associated with attenuated joint pathology in humans [22]. There could also be advantages of the opal stop mutation dependent upon the nature of the host and geographical constraints [28]. Secondly, the G82R mutation (that occurred in 17% of the sequenced challenge stock) is recognised as an attenuating mutation. The G82R attenuation is associated with altered glycosaminoglycan binding, reduces in vivo replication, altered the establishment of viremia, altered the activation of early inflammatory responses in mosquitos, zebrafish [23] and a mouse model [25], and has also been exploited for vaccine development [29]. Both mutations are present in the stock at relatively low levels, and we demonstrate that the replication of the virus appeared unimpaired in vivo in control macaques attaining an expected log10 7–9 CHIKV RNA IU/mL two days post-challenge, which appears characteristic of the wild-type CHIKV challenge in similar naïve NHP challenge studies [12,16,30,31,32]. Finally, it should be noted that the stock of virus used in this study is identical with that used to evaluate the Valneva vaccine [31]. The data from that study were used to secure regulatory approval for that vaccine in USA, Europe, Canada and the UK.

Following challenge, there was a statistically significant higher (>7–9 log10) viral RNA burden in the plasma of placebo controls compared with BBV87-immunised animals, and no infectious virus could be recovered from vaccinated animals. Virus levels in serum or the plasma of control animals detected by RT-qPCR and infectious culture appeared consistent with other published data [32]. Analyses of lymphoid tissues at necropsy on day 14–15 (a time when viremia was suppressed in control macaques) revealed that 18 of 20 lymphoid tissues sampled amongst the five challenged controls had detectable CHIKV RNA, whereas only a single tissue sample in one vaccinated animal had a low positive signal by RT-qPCR. Intriguingly, the positive tissue in the vaccinated macaque was the draining lymph node for the site of intradermal inoculation of the virus challenge.

In addition to detectable virus in blood and persistence in selected lymphoid tissues, control macaques exhibited a number of inflammatory and pathological responses associated with chikungunya virus infection. These included a marked disruption of thermoregulation coinciding with the peak viremia in the plasma, as well as transient increased levels of cytokines Interleukin 1 Receptor agonist (IL-1ra), Monocyte Chemoattractant Protein-1 (MCP-1) and C-reactive protein (CRP). Elevated CRP has been identified as a marker associated with CHIKV disease and has been used to monitor disease [33]. The cytokine concentrations at two days post-challenge correlated with peak viraemia in an apparent dose-dependent manner. By contrast, in BBV87-immunised macaques, no significant perturbation of diurnal temperature profile or in cytokine levels were observed following challenge. The temperature increase observed in control animals was not as high as that observed by others, and this may be due to the monitoring method applied. The surgical implantation of a telemetry device subcutaneously enabled continuous temperature monitoring throughout the challenge period rather than measurement at a single point when under anaesthesia [16]. In relation to modelling chikungunya’s long-term arthralgia, of particular interest was the identification of subtle but significant histopathological changes in finger joints from control infected macaques at days 14–15, and areas of inflammation in the synovium and accompanying tissues were detected. Whilst areas of similar pathology were also noted in equivalent joints in vaccinated macaques, independent “blinded” scoring by two veterinary pathologists judged that these inflammatory changes were significantly lower amongst the immunised, virus-challenged macaques as compared to placebo-treated, virus challenged animals. This is the first report of the detection of the increased frequency of early inflammation in the joint tissue of cynomolgus macaques following infection with this dose of chikungunya virus. It warrants further studies to establish whether these changes are progressive with time as most NHP studies involving the chikungunya virus are relatively short compared with the timescale of the long-term debilitating joint pain that accompanies some clinical CHKV infections. It would also warrant investigation as to whether other challenge models of CHIKV may exhibit more marked pathology.

Overall, this study demonstrates that active immunisation with 20 µg dose BBV87 vaccine protects against both chikungunya virus infection and disease in an otherwise susceptible host. The lack of inflammation and pathology after challenge may not be unexpected since there was no evidence of an anamnestic anti-CHIKV IgG response following challenge in immunised macaques, whereas, in control macaques, marked IgM and IgG responses were detected two weeks after the virus challenge. This suggests that the immunisation protocol had prevented any detectable viral replication upon challenge (i.e., “sterilising immunity”) against the LR2006 OPY1 virus, which is an appropriate challenge virus to evaluate a vaccination protocol designed to be effective against viruses circulating in SE Asia and other countries bordering the Indian Ocean.

Others have established cynomolgus macaque models to study the efficacy of live attenuated chikungunya virus vaccines. Rossi et al. [32] evaluated protection following active immunisation with a viral vectored vaccine, and Roques et al. [31] evaluated whether immune sera from clinical vaccine volunteers could confer passive protection. In both of these studies, as is the case in our study, the focus was on the measurement of serological responses prior to challenge, the detection and measurement of virus in blood and tissues, and the systemic markers of inflammation and disease. Our additional inclusion of a more detailed histopathological analysis of joint tissues is a novel observation worthy of corroboration by others. Spontaneous cases of arthritis and associated inflammation have been reported in approximately 20% of free-ranging rhesus macaques with a lower incidence in laboratory NHPs [34]. No bone-related changes attributable to infection were observed; only artefactual changes, possibly due to fixation processes including decalcification, were noted. The use of a well-characterized challenge virus stock as described in this report will facilitate the comparison of the relative value of different treatments to control infection and disease, and it should be noted that the challenge stock and challenge dose used in our study were identical to those used by Roques et al. However, it should be noted that the vaccine used to immunise clinical trial volunteers was a clone derived from the challenge stock.

The future development of the BBV87 vaccine will be the establishment of correlates and the mechanism of vaccine protection against infection and/or disease. The establishment of these markers will allow clinical trial design to be optimised, including the choice of trial endpoints. The recent demonstration by Roques et al. [31] of titratable protection following the transfer of clinical trial immune serum demonstrates the role for serological responses alone in vaccine protection. Similar protection has also been achieved with mouse challenge models with vaccination and the passive transfer of serum from vaccinated mice [35,36]. This concurs with sero-epidemiological studies that acquired protection against symptomatic CHIKV infection and disease correlates anti-chikungunya neutralising activity [37]. It has been demonstrated in a mouse model that a circulating NT_50_ of 35 of mouse vaccinee sera was required for protection against CHIKV challenge [38]. Clearly our study indicates that a neutralising antibody response of 100 NT_50_ was associated with the robust protection observed. Nevertheless, this active immune protection could also be attributed to a cellular response following vaccination. Further pre-clinical studies would be required to dissect the relative contribution of serological and cellular responses following vaccination with 20 µg BBV87. Notwithstanding, these data suggest that 20 µg BBV87 is a potent vaccine. A comparison of the responses obtained from clinical studies with those obtained in this NHP model would be illuminating. Furthermore, serum transfer studies that would exclude a role for cellular responses could establish an antibody cut-off for protection that would inform clinical development and facilitate pathways to regulatory approval of this and other experimental CHIKV vaccines.

## 5. Conclusions

The pre-clinical evaluation of the 20 µg BBV87 whole virus-inactivated vaccine in a cynomolgus macaque model of infection established its ability to generate serological immune responses, including neutralising antibodies. Following intra-dermal challenge with an epidemiologically relevant wild-type clinical isolate, apparent sterilising protection was observed. This study paves the way for more detailed studies to determine the level and nature of protective correlates generated with this vaccine against the chikungunya virus.

## Figures and Tables

**Table 1:** Summary of NGS analysis showing location of any changes from reference sequence with >500 reads and >5% occurrence (LR2006 OPY1, complete genome, NCBI accession DQ443544.2).

Position (nt).	Ref	Alt	Proportion (% Alt, Ivar)	Total Reads	Coding	Location	Comments
20	G	A	6	2602	-	5′ UTR	n/a
20	G	C	22	3181	-	5′ UTR	n/a
22	A	G	11	3654	-	5′ UTR	n/a
24	C	T	21	3977	-	5′ UTR	n/a
995	CAC	TAC	76	12,524	H to Y	non-structural polyprotein	n/a
1052	R (A or G)TG	GTG	100	12,186	-	non-structural polyprotein	no change, already a possible nt in complete genome sequence
1445	TCG	CCG	8	11,478	S to P	non-structural polyprotein	n/a
4167	GRC (A or G)	GAC	98	23,828	-	non-structural polyprotein	no change, already a possible nt in complete genome sequence
5049	AKA (G or T)	AGA	96	20,962	-	non-structural polyprotein	no change, already a possible nt in complete genome sequence
5645	TGA	CGA	6	24,158	Stop to R	non-structural polyprotein	opal stop mutation [22,23]
6505	GAA	GAT	100	2926	E to D	non-structural polyprotein	n/a
8552	GAC	GGC	11	21,761	D to G	structural polyprotein	downstream of furin cleavage site between E3 and E2 [24]
8785	GGG	CGG	17	14,143	G to R	structural polyprotein	G82R mutation in E2 glycoprotein, recognised as attenuating [23,25]

## Data Availability

The sequencing data obtained for the challenge stock are available from the SRA Data Repository.

## Supplementary Materials

The following supporting information can be downloaded at: https://www.mdpi.com/article/10.3390/v17040550/s1, Table S1: Primer pair sequence and location relative to CHIKV genome (Genbank: DQ443544.2); Table S2: Haematological data showing group means and individual results for blood analyses. Table S3: Joint pathology scores received from Veterinary Pathologists for each parameter and animal and each location. Score of 0 denotes no lesion or inflammation, 1: mild injury or inflammation or 2: significant injury or inflammation.
