# Supplementary material for: Inactivated Viral Vaccine BBV87 Protects Against Chikungunya Virus Challenge in a Non-Human Primate Model
Source: Viruses. 2025 Apr 10;17(4):550. doi: 10.3390/v17040550 (PMC12031606; doi:10.3390/v17040550)
Supplement: Supplementary file 1 [file viruses-17-00550-s001.zip › viruses-3544961-supplementary.pdf]

Supplementary Table 1: Primer pair sequence and location relative to CHIKV genome (Genbank: DQ443544.2).

Name	Sequence	Direction	Position	Size
ChikVPS1-P1-For	ATGGCTGCGTGAGACACAC	Forward 1	1-19	~ 3.1 kb
ChikVPS1-P1-Rev	TGAGACCACTGCCTATCATTTA	Reverse 1	3162-3183	
ChikVPS1-P21-For	GACCTTCGATACATTCCAAA	Forward 2	3079-3098	~3.4 kb
ChikVPS1-P2-Rev	GAACCTATCCATTGGTACATC	Reverse 2	6491-6511	
ChikVPS1-P3-For	ACACACTACAGAATGTACTGGCA	Forward 3	6243-6265	~2.6 kb
ChikVPS1-P3-Rev	CGTACATGAGTGACTAATCTTCCT	Reverse 3	8893-8916	
ChikVPS1-P4-For	CAGCACCGTGTACGATTACTGG	Forward 4	8804-8825	~3.0 kb
ChikVPS1-P4-Rev	GTTCGGAGAATCGTGGAAGAGTTC	Reverse 4	11747-11770	

Supplementary Table 2: Haematological data showing group means and individual results for blood analyses.

		Day 0				Day 1				Day 2				Day 3			
		Vaccinated		Control		Vaccinated		Control		Vaccinated		Control		Vaccinated		Control	
ID		Mean	SEM	Mean	SEM	Mean	SEM	Mean	SEM	Mean	SEM	Mean	SEM	Mean	SEM	Mean	SEM
	RBC (x1012/L) 5.2-7.5	6.20	0.21	6.14	0.43	6.15	0.22	5.78	0.61	6.15	0.25	5.64	0.43	6.03	0.22	5.56	0.42
	Hb (g/dl) 10.3-13.9	11.50	0.53	11.20	1.43	11.42	0.51	10.58	1.61	11.40	0.52	10.22	1.35	11.08	0.50	10.12	1.37
	HCT (PCV) 34-46	37.78	1.22	36.90	3.65	37.30	1.20	34.74	4.62	37.36	1.12	33.94	3.63	36.48	1.11	33.02	3.49
	MCV (fL) 54-71.2	61.20	2.84	60.00	3.39	60.80	2.75	59.40	3.54	60.80	2.75	59.60	3.57	60.80	2.75	59.20	3.37
	MCH (pg) 16.1-21.8	18.58	1.00	18.02	1.54	18.66	1.07	17.94	1.51	18.58	1.00	17.86	1.52	18.50	1.01	17.90	1.50
	MCHC (g %) 28.0-32.7	30.32	0.60	29.94	1.10	30.56	0.64	30.04	0.93	30.42	0.60	29.72	0.99	30.40	0.64	30.18	1.09
	RDW %	14.98	0.49	14.94	0.69	14.78	0.36	14.36	0.79	15.04	0.38	15.02	0.75	14.74	0.44	14.48	0.70
	WBC (X109/L) 3.5-14.7	9.10	0.88	10.02	1.36	8.38	0.96	7.88	1.13	8.30	1.35	4.94	0.72	7.66	1.06	6.18	0.91
N'phils	%	59.34	11.05	64.44	5.78	49.76	9.40	61.08	5.15	42.12	8.90	55.80	3.23	39.76	8.82	46.46	5.81
N'phils	(X109/L) 0.1-6.9	5.57	1.12	6.37	0.87	4.26	1.10	4.76	0.80	3.58	0.92	2.77	0.45	2.91	0.70	2.88	0.56
L'cytes	%	34.94	10.24	28.14	5.21	43.88	8.65	30.68	4.99	51.80	8.39	29.82	2.84	53.74	8.24	40.80	5.98
L'cytes	(X109/L) 0.8-9.7	3.02	0.74	2.92	0.81	3.60	0.78	2.43	0.59	4.22	0.97	1.49	0.28	4.24	1.03	2.55	0.62
M'cytes	%	4.46	0.92	5.66	0.75	5.14	0.75	6.86	0.89	4.70	0.56	13.08	1.67	5.16	0.49	11.26	1.62
M'cytes	(X109/L) up to 1.2	0.41	0.11	0.48	0.14	0.43	0.07	0.56	0.10	0.40	0.08	0.62	0.07	0.40	0.08	0.66	0.07
E'phils	%	0.82	0.08	1.22	0.34	0.82	0.17	1.04	0.21	0.94	0.12	1.08	0.19	0.92	0.23	1.10	0.25
E'phils	(X109/L) up to 0.7	0.08	0.01	0.13	0.04	0.07	0.02	0.09	0.02	0.07	0.01	0.05	0.01	0.07	0.02	0.06	0.02
B'phils	%	0.44	0.02	0.54	0.18	0.40	0.04	0.34	0.04	0.44	0.06	0.22	0.04	0.42	0.07	0.40	0.05
B'phils	(X109/L) up to 0.3	0.04	0.00	0.05	0.01	0.03	0.01	0.03	0.01	0.04	0.01	0.01	0.00	0.03	0.01	0.02	0.01
P'lets	(X109/L) 157-498	363.40	22.12	335.20	49.54	347.40	34.64	264.80	54.69	310.60	38.52	231.40	33.14	305.60	35.88	242.20	37.94
MPV	%	8.34	0.28	8.48	0.10	8.28	0.35	8.56	0.09	8.52	0.31	8.52	0.23	8.44	0.45	8.42	0.08

		Day 4				Day 6				Day 9				Day 14			
		Vaccinated		Control		Vaccinated		Control		Vaccinated		Control		Vaccinated		Control	
ID		Mean	SEM	Mean	SEM	Mean	SEM	Mean	SEM	Mean	SEM	Mean	SEM	Mean	SEM	Mean	SEM
	RBC (x1012/L) 5.2-7.5	4.28	0.32	3.83	0.38	5.74	0.24	5.30	0.43	5.56	0.23	5.34	0.44	5.49	0.24	5.25	0.42
	Hb (g/dl) 10.3-13.9	10.82	0.50	9.90	1.30	10.76	0.56	9.74	1.34	10.28	0.40	9.68	1.32	10.04	0.67	9.28	1.22
	HCT (PCV) 34-46	28.40	1.14	25.14	2.57	34.74	1.30	31.54	3.37	33.10	0.74	31.34	3.39	32.82	1.49	30.64	3.23
	MCV (fL) 54-71.2	67.20	3.61	65.80	4.44	60.80	2.67	59.00	3.03	60.00	2.61	58.20	3.02	60.00	2.47	57.80	3.25
	MCH (pg) 16.1-21.8	26.14	2.74	26.08	3.22	18.84	1.04	18.06	1.48	18.58	1.01	17.82	1.42	18.30	1.00	17.42	1.48
	MCHC (g %) 28.0-32.7	38.52	2.64	39.14	2.65	30.96	0.61	30.40	1.21	30.98	0.67	30.38	1.10	30.50	0.75	29.82	1.06
	RDW %	13.12	0.45	13.70	1.46	14.80	0.40	14.18	0.73	14.54	0.48	14.52	0.79	14.86	0.45	14.74	0.55
	WBC (X109/L) 3.5-14.7	8.90	1.85	5.80	1.09	10.06	1.66	8.14	2.30	7.54	0.98	6.78	0.78	8.80	1.60	8.12	0.86
N'phils	%	42.20	8.94	41.58	7.00	48.62	11.32	41.82	10.32	40.44	8.63	30.02	5.28	31.72	7.78	30.13	5.69
N'phils	(X109/L) 0.1-6.9	3.78	1.17	2.30	0.39	5.18	1.77	3.85	1.57	2.94	0.62	1.96	0.29	2.45	0.40	2.50	0.56
L'cytes	%	50.46	8.45	44.48	6.98	44.62	10.67	43.92	8.64	52.18	8.00	56.30	5.80	60.02	7.35	55.82	6.90
L'cytes	(X109/L) 0.8-9.7	4.45	1.11	2.66	0.81	4.19	1.15	3.39	1.26	4.04	0.98	3.91	0.76	5.61	1.58	4.52	0.77
M'cytes	%	5.94	0.60	12.52	1.12	5.36	0.69	11.56	2.69	5.74	0.71	10.96	1.14	5.64	0.46	6.72	0.63
M'cytes	(X109/L) up to 1.2	0.56	0.15	0.73	0.16	0.53	0.11	0.72	0.11	0.42	0.06	0.71	0.04	0.51	0.11	0.53	0.04
E'phils	%	0.98	0.24	0.91	0.28	1.06	0.21	2.14	0.80	1.20	0.25	1.78	0.29	2.18	0.65	1.70	0.14
E'phils	(X109/L) up to 0.7	0.08	0.02	0.06	0.02	0.10	0.02	0.12	0.02	0.10	0.03	0.13	0.03	0.18	0.05	0.14	0.03
B'phils	%	0.40	0.08	0.38	0.07	0.34	0.04	0.56	0.13	0.44	0.09	0.94	0.20	0.44	0.07	0.42	0.04
B'phils	(X109/L) up to 0.3	0.04	0.02	0.02	0.01	0.04	0.01	0.04	0.01	0.03	0.01	0.06	0.01	0.04	0.01	0.03	0.01
P'lets	(X109/L) 157-498	235.60	35.93	175.20	18.55	341.80	35.74	279.20	50.91	338.80	47.45	479.60	207.68	378.00	41.98	561.40	189.73
MPV	%	8.78	0.37	9.90	1.13	8.36	0.29	8.86	0.27	8.44	0.18	8.90	0.25	8.42	0.33	8.84	0.28

Supplementary Table 3: Joint pathology scores received from Veterinary Pathologists for each parameter and animal and each location. Score of 0 denotes no lesion or inflammation, 1: mild injury or inflammation or 2: significant injury or inflammation.

	S50 (Vaccinated)									
**Location**	**R thumb**	**R thumb kn**	**RF1**	**RF1 Kn**	**RF2**	**RF2 Kn**	**RF3**	**RF3 Kn**	**RF4**	**RF4 Kn**
**1. Synovium**	0	0	0	0	0	0	0	0	0	0
**2. Articular cartilage**	0	0	0	0	0	0	0	0	0	0
**3. Skel mucle**	0	0	0	0	0	0	0	0	0	0
**4. Periosteum**	0	0	0	0	0	0	0	0	0	0
**5. Cortical bone**	0	0	0	0	0	0	0	0	0	0
**Total score**	0	0	0	0	0	0	0	0	0	0
	**S51 (Vaccinated)**									
**Location**	**R thumb**	**R thumb kn**	**RF1**	**RF1 Kn**	**RF2**	**RF2 Kn**	**RF3**	**RF3 Kn**	**RF4**	**RF4 Kn**
**1. Synovium**	0	0	0	0	0	0	0	0	0	1
**2. Articular cartilage**	0	0	0	0	0	0	0	0	0	0
**3. Skel mucle**	0	1	0	1	0	0	0	0	0	0
**4. Periosteum**	0	0	0	0	0	0	0	0	0	0
**5. Cortical bone**	0	0	0	0	0	0	0	0	0	0
**Total score**	0	1	0	1	0	0	0	0	0	1
	**S54 (Vaccinated)**									
**Location**	**R thumb**	**R thumb kn**	**RF1**	**RF1 Kn**	**RF2**	**RF2 Kn**	**RF3**	**RF3 Kn**	**RF4**	**RF4 Kn**
**1. Synovium**	0	1	1	0	0	1	1	0		
**2. Articular cartilage**	0	1	1	0	0	0	1	0		
**3. Skel mucle**	0	0	0	0	0	0	0	0		
**4. Periosteum**	1	2	2	0	0	2	1	2		
**5. Cortical bone**	0	1	2	0	0	2	0	2		
**Total score**	1	5	6	0	0	5	3	4		
	**S58 (Vaccinated)**									
**Location**	**R thumb**	**R thumb kn**	**RF1**	**RF1 Kn**	**RF2**	**RF2 Kn**	**RF3**	**RF3 Kn**	**RF4**	**RF4 Kn**
**1. Synovium**	0	0	0	0	1	1	0	0	0	1
**2. Articular cartilage**	0	0	0	0	0	0	0	0	0	0
**3. Skel mucle**	0	0	0	0	0	0	0	0	0	0
**4. Periosteum**	0	0	0	0	1	1	0	0	0	0
**5. Cortical bone**	0	0	0	0	1	1	0	0	0	0
**Total score**	0	0	0	0	3	3	0	0	0	1
	**S59 (Vaccinated)**									
**Location**	**R thumb**	**R thumb kn**	**RF1**	**RF1 Kn**	**RF2**	**RF2 Kn**	**RF3**	**RF3 Kn**	**RF4**	**RF4 Kn**
**1. Synovium**	1	0	1	0	0	0	0	0	2	0
**2. Articular cartilage**	0	1	0	0	0	0	0	0	2	0
**3. Skel mucle**	0	1	0	0	0	0	0	0	0	0
**4. Periosteum**	0	1	0	1	2	0	2	0	1	0
**5. Cortical bone**	0	1	0	1	2	0	2	0	0	0
**Total score**	1	4	1	2	4	0	4	0	5	0

	**S49 (Control - CHIKV only)**									
**Location**	**R thumb**	**R thumb kn**	**RF1**	**RF1 Kn**	**RF2**	**RF2 Kn**	**RF3**	**RF3 Kn**	**RF4**	**RF4 Kn**
**1. Synovium**	0	0	0	0	0	0	0	0	0	0
**2. Articular cartilage**	0	0	0	0	0	0	0	0	0	0
**3. Skel mucle**	0	0	0	1	0	0	1	0	0	0
**4. Periosteum**	0	0	0	0	0	0	0	0	0	0
**5. Cortical bone**	0	0	0	0	0	0	0	0	0	0
**Total score**	0	0	0	1	0	0	1	0	0	0
	**S52 (Control - CHIKV only)**									
**Location**	**R thumb**	**R thumb kn**	**RF1**	**RF1 Kn**	**RF2**	**RF2 Kn**	**RF3**	**RF3 Kn**	**RF4**	**RF4 Kn**
**1. Synovium**	0	0	0	0	1	1	0	1	1	1
**2. Articular cartilage**	0	0	0	0	1	0	0	0	0	0
**3. Skel mucle**	0	0	1	0	0	0	1	1	0	1
**4. Periosteum**	0	0	0	0	0	0	2	1	0	2
**5. Cortical bone**	0	0	0	0	0	0	0	0	0	1
**Total score**	0	0	1	0	2	1	3	3	1	5
	**S55 (Control - CHIKV only)**									
**Location**	**R thumb**	**R thumb kn**	**RF1**	**RF1 Kn**	**RF2**	**RF2 Kn**	**RF3**	**RF3 Kn**	**RF4**	**RF4 Kn**
**1. Synovium**	0	0	0	2		0	1	1		
**2. Articular cartilage**	0	0	0	2		0	0	0		
**3. Skel mucle**	0	0	0	0		0	1	0		
**4. Periosteum**	0	0	2	0		0	2	0		
**5. Cortical bone**	0	0	2	0		0	2	0		
**Total score**	0	0	4	4		0	6	1		
	**S56 (Control - CHIKV only)**									
**Location**	**R thumb**	**R thumb kn**	**RF1**	**RF1 Kn**	**RF2**	**RF2 Kn**	**RF3**	**RF3 Kn**	**RF4**	**RF4 Kn**
**1. Synovium**	1	2	1	0	1	0	2	0	1	0
**2. Articular cartilage**	0	1	0	0	1	0	1	0	1	0
**3. Skel mucle**	0	0	0	0	0	0	0	0	1	0
**4. Periosteum**	0	1	2	0	1	0	2	0	2	1
**5. Cortical bone**	0	1	2	0	1	0	2	0	2	1
**Total score**	1	5	5	0	4	0	7	0	7	2
	**S57 (Control - CHIKV only)**									
**Location**	**R thumb**	**R thumb kn**	**RF1**	**RF1 Kn**	**RF2**	**RF2 Kn**	**RF3**	**RF3 Kn**	**RF4**	**RF4 Kn**
**1. Synovium**		0	1	0	0	0	1	0	1	0
**2. Articular cartilage**		0	0	0	0	0	0	0	0	0
**3. Skel mucle**	0	0	0	0	0	0	0	0	0	0
**4. Periosteum**		0	0	2	0	1	0	0	1	1
**5. Cortical bone**		0	0	2	0	1	0	0	1	1
**Total score**	0	0	1	4	0	2	1	0	3	2
